# A Bioscience Educators’ Purpose in a Modern World

**DOI:** 10.1523/ENEURO.0364-25.2025

**Published:** 2025-11-05

**Authors:** Connie Pritchard, Sean Hubbert, Emma Yhnell

**Affiliations:** ^1^School of Biosciences, Cardiff University, Cardiff CF10 3AX, United Kingdom; ^2^School of Biological and Medical Sciences, Oxford Brookes University, Oxford OX3 0BP, United Kingdom

**Keywords:** belonging, digital learning, educator, innovation, pedagogy, purpose

## Abstract

Higher education (HE) is undergoing rapid transformation, shaped as a result of the COVID-19 pandemic, the expansion of digital learning, and the increasing presence of artificial intelligence (AI). For educators, these shifts raise important questions about their evolving purpose and responsibilities. In this commentary, we reflect on the role of bioscience educators in the United Kingdom, highlighting the enduring need for human connection, empathy, and belonging in teaching, alongside the integration of digital tools. We discuss changing student motivations, the necessity of flexible and inclusive learning environments, and the balance between traditional practices and innovative pedagogies. Practical training, active learning, and responsible engagement with emerging technologies remain central to equipping students with transferable skills such as adaptability, critical thinking, and resilience. We argue that while digital innovations can enhance accessibility and engagement, they cannot replace the uniquely human dimensions of teaching. Ultimately, bioscience educators must embrace their dual role as facilitators and lifelong learners, modeling curiosity, vulnerability, and inclusivity to empower students to thrive in an increasingly complex world.

## Graphical abstract

**Figure:**
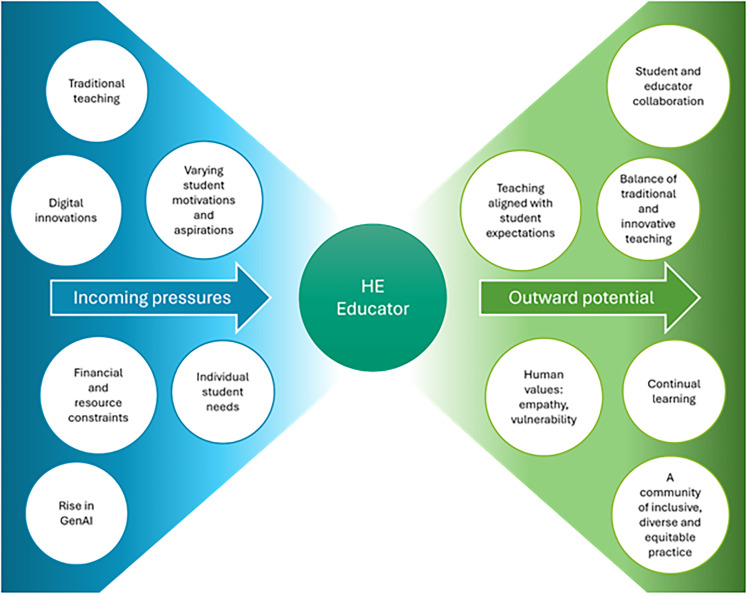
Visual Abstract

## Introduction

As educators working across the United Kingdom, at different career stages and within different types of higher education institutions (HEIs), we have been reflecting on our respective teaching roles and what the future may hold for them. Recent years have brought dramatic shifts to higher education (HE): the repercussions of the COVID-19 pandemic (Burki, 2020; Burns et al., 2020; Kazemian and Grant, 2024), the rapid emergence of digital learning (Chatterton, 2022; Walker and Voce, 2023), and the rise of artificial intelligence (AI; Bearman et al., 2023; Crompton and Burke, 2023), which are all significant for HE.

During the pandemic-driven shift to online education, many educators reported that their greatest concerns related to maintaining meaningful connection, equity, and emotional support for students (Graham et al., 2024), highlighting the deeply relational and human dimensions of teaching in HE. At the same time, learner expectations and the lived realities of studying are evolving; the UK HE funding model means that many learners are juggling employment, caring responsibilities, or other commitments alongside their studies (Taylor, 2025). Although access to information has never been easier, the abundance of resources available does not always translate into accurate, trusted knowledge, nor meaningful or equitable learning experiences for students (Amjad et al., 2024).

HEIs themselves are under increasing strain, balancing the demands of widening participation, quality assurance, and graduate outcomes with persistent financial pressures. Many UK universities are now reporting deficits and undertaking restructuring and redundancy programs (University and College Union [UCU], 2025 https://qmucu.org/qmul-transformation/uk-he-shrinking/).

In this commentary, we reflect on the role that educators in HE can and should play in 2025 and beyond, with a particular focus on bioscience education. We ask: what do learners truly need from their educators in order to thrive in a complex, rapidly changing world? While the tools and contexts of teaching will continue to evolve, we argue that the central purpose of the educator remains clear: to be human. That is, to model empathy, to foster belonging, and to prepare learners not only to become subject-specific experts but also to develop transferable skills of adaptability, resilience, lifelong learning, and the ability to critique, question, and analyze information. Digital innovations and AI can assist in educational delivery and the generation of knowledge, but they cannot (yet) replace the uniquely human relational qualities that are central to the student experience and to teaching and learning in HE.

## Motivations for Learning: Responding to Learner Needs in a Digital World

Considering the unique motivations that learners have for studying is an important factor in understanding their individual and specific learning journeys. Within the biosciences, students’ motivations are varied and nuanced; some aim primarily to gain specific career-focused skills and knowledge, and some choose biosciences purely out of interest, while others expect that they will be academically successful in a specific bioscience course (Hsu and Dudley, 2022; Dai et al., 2025). Upon graduation, students who study a bioscience degree do not necessarily proceed into a career as a practicing scientist (HESA, 2025). Therefore, individual student motivations for both choosing a bioscience course and their aspirations upon graduation are important considerations to ensure that educational practices and student expectations are aligned.

In addition, there are notable changes in how students access learning, and the environment in which they learn most effectively as a result of increases in digital education provision. The marked shift in the accessibility of digital learning tools will inevitably affect the role educators play in creating learning environments. Digital learning tools can be viewed as either internal, such as session recordings, or external, such as YouTube and, more recently, AI large language models such as ChatGPT. The growth in the latter is particularly stark, with 92% of students now utilizing AI in some form to facilitate learning (HEPI, 2025). The wide-ranging benefits of the use of digital learning have become relatively clear. Blended learning approaches, a combination of traditional face-to-face teaching and online learning, are popular, and many students feel these approaches benefit their learning (Tahir et al., 2022). Furthermore, the flexibility in accessing learning allows students to learn at their own pace while also providing a more inclusive environment for students who may be restricted in their availability to attend in-person sessions due to caring responsibilities or paid-work commitments. This highlights the need to adopt a flexible and adaptable learning environment that allows all learners to engage equitably and fully with their bioscience course.

Despite the accessibility of information and digital learning tools, we believe that educators still play an important and necessary role in student learning. Although external digital learning tools are increasingly accessible, in-session recordings from educators themselves are also popular among students (Voelkel et al., 2023). Although reduced in-person session attendance may be of frustration to some educators due to perceived lack of student engagement, it is critical to appreciate that in-person attendance does not necessarily guarantee meaningful student engagement in learning (Eika, 2021), and there may be a range of reasons which explain the lack of in-person attendance.

However, many students benefit greatly from attending in-person teaching sessions; they feel more engaged, it makes them accountable, and they value the social integration that in-person teaching provides (Mulvihill and Martin, 2023). Moreover in the biosciences, learning is not limited to theoretical knowledge; large components of the course and the associated skills developed are taught in a variety of practical classes. This fundamental component of bioscience courses relies heavily on effective in-person training by the educator, such as the practical and tactile skills including learning how to use a pipette, how to plate agar, and how to use a microscope. Virtual lab simulations can support in-person practical teaching; they allow students to practice techniques without real-world consequences, and they can help to improve lab confidence and reduce anxiety (George-Williams et al., 2022; Racey et al., 2024) and improve grades in summative assessment (Cassambai et al., 2022), but crucially, they are designed to supplement in-person practical teaching, rather than replace it.

Educators must evolve, adapt, and embed digital learning tools to enhance the student experience and embrace alternative teaching methods within their practice. Digital resources can be utilized to allow students to learn more content independently from the formal learning environment, freeing up more contact time to actively explore educational content within in-person sessions. More active teaching methods have not only been shown to be more effective for student learning than traditional passive methods (Freeman et al., 2014) but may also provide greater opportunity for learners to interact with both their peers and educators in an nondidactic environment. Moreover, active teaching methods help to foster learning environments that not only emphasize the learning of information but promote improving broader transferable skills such as communication, teamwork, critical thinking, problem-solving, and creativity, as well as improving students’ sense of belonging within their cohort.

## Innovations in Education: Recognizing the Barriers and Findings Solutions

There are many barriers to providing innovative education in HE, and it is often an active and ongoing choice for educators in HE across the United Kingdom to take on the challenge of innovation in education. However, in order to remain current and interesting to growing numbers of learners, educators must ensure that their educational content is relevant and engaging to diverse groups of learners. However, a significant barrier to educational innovation is the understandable tension between maintaining the familiarity and consistency of traditional methods and embracing novel, sometimes experimental approaches which take time, thought, and creativity. This tension can lead to risk-aversion and loss of potentially valuable learning experiences; therefore, educators must consciously balance respect for established practices with a willingness to innovate, creating space within the curriculum to pilot and evaluate novel methods. With varying and numerous academic responsibilities, the time and resources required to innovate in education should not be underestimated.

An additional challenge, particularly in the United Kingdom, is the increase in overall student numbers (Giannakis and Bullivant, 2016, Hewitt, 2020). This presents difficulty in maintaining meaningful student–educator relationships, fostering individual feelings of belonging and delivering personalized support. In some cases, large classes may be taught online due to lack of sufficient teaching spaces. It is vital for educators to actively explore and integrate practical strategies into their teaching to mitigate these issues and to do whatever they can to address them. While there is no simple solution, online quiz tools or audience response systems such as Mentimeter can encourage interactive engagement even in large (Mohin et al., 2022) and online classes (Armstrong et al., 2024), providing a voice for students who might otherwise remain silent.

We encourage educators to actively seek to understand how students are already engaging with new technologies, such as AI and digital learning tools, and to have open and honest conversations about how these can be responsibly integrated into teaching methodologies and assessments. Collaborating with students as partners or cocreators of learning experiences ensures educational relevance, overcomes problematic power dynamics, encourages responsible digital literacy, and supports their sense of belonging and inclusion (Ali et al., 2021; Gregory-Ellis, 2022).

## Belonging and Inclusion: Creating a Culture of Empowerment

Creating a genuine culture of belonging and inclusion within the educational environment requires actively addressing power dynamics, particularly those traditionally inherent within educator–student relationships (Mercer-Mapstone et al., 2018; Nakonechna and Schuetz, 2025). Educators may and can consciously seek ways to reduce hierarchical barriers by presenting themselves as facilitators rather than solely authoritative figures. For example, strengthening personal tutoring systems and pastoral support can ensure students have at least one direct, supportive human relationship with faculty, thereby reinforcing their sense of belonging and identity. Additionally, proactively facilitating informal spaces and opportunities for student gatherings, such as study groups, academic cafes, or peer-led workshops, can enhance community cohesion, student well-being and student satisfaction (Waldock et al., 2016; Geister et al., 2025).

A fundamental value of educators is that we are human: we make mistakes, we do not know everything, and we each have a unique set of experiences, both within our professional and personal lives, that have the power to influence how we deliver education. While expressing this vulnerability as an educator may be uncomfortable, embracing vulnerability as a pathway to generating deeper connections with students and enabling personal growth within HE can be empowering for both educator and student alike (Christodoulidi, 2024). Sharing carefully selected personal examples which demonstrate individuality, identity, and vulnerability during teaching sessions can be powerful and memorable for students. Demonstrating this vulnerability alongside empathy and realism invites students to embrace alternative perspectives and cultivates an environment where differences in thinking are valued and encouraged. Facilitating respectful dialogue around challenging or sensitive topics must be thoughtfully and carefully integrated into teaching sessions to ensure that all views are expressed and heard and that mutual respect remains intact, even when opinions can diverge significantly. Not all students, nor staff, will know how to respectfully query or disagree, so clear behavioral expectations and boundaries should be established early on to facilitate a mutual understanding of acceptable and appropriate conduct.

Paying particular and careful attention to equity, diversity, and inclusion practices is essential, as students from underrepresented or marginalized backgrounds may disproportionately experience exclusion, misunderstanding, or discomfort in HE (Howard and Barnett, 2015). Educators should proactively recognize, manage, and mitigate behaviors that inadvertently disadvantage certain groups, ensuring equity is consistently prioritized in education. By intentionally incorporating culturally diverse examples, materials, and perspectives into teaching content, educators can validate varied student identities, fostering a stronger sense of belonging. Such inclusive practices enhance the learning experience, enrich classroom discussions, and encourage students to authentically represent and be themselves in educational settings.

Mutual respect underpins the entire framework of an inclusive educational environment. Students must be assured their voices matter and implementing clear channels for feedback, such as surveys, reflective discussions, or student representation panels, is imperative. Equally important is visibly reflecting on, responding to, and transparently communicating actions (or limitations) arising from feedback which is sought. Closing the feedback loop, even when not all suggestions can be accommodated, ensures that students genuinely feel heard and respected, even if they do not necessarily agree with the actions taken or decisions made.

A sense of belonging and inclusion is foundational to student empowerment. While not all students actively seek empowerment or wish to assume roles of agency, institutions must create opportunities and safe spaces (both physically and psychologically) for students to take on empowered roles if and when they choose (EUA, 2025). Pastoral support, through the role of personal tutors, student’s advice services, and/or counseling services, also plays a vital role. As Maslow theorizes, in order for one to achieve their potential, their physical and psychological needs must be recognized, respected, and addressed (Maslow, 1943).

## Always Learning: Educators Are Lifelong Learners

To be effective and engaging educators within the biosciences in HE, we must commit to authentically modeling lifelong learning to others through our own teaching approaches. We must model to learners that curiosity, adaptability, and humility are central to professional and personal growth (Candy, 2002). Educators may well be subject-specific experts in their fields or disciplines, but it is still not realistic or human to be able to know absolutely everything about a subject or discipline. The dual and authentic role of educator and continual learner enhances teaching provision and strengthens credibility in HE teaching and learning environments while also reinforcing the idea that learning is never complete, even for those teaching at the highest levels. Therefore, we encourage educators to be open and honest with learners, it is absolutely the right thing to do to admit if you do not know the answer, before following up with a commitment to find out and get back to the learner at a later date.

## Conclusion

In a rapidly changing world, educators cannot remain static repositories of knowledge delivered in unengaging, didactic ways. In the biosciences, discoveries evolve continually, and so too must the pedagogies that underpin teaching. By embracing the opportunities and challenges of emerging technologies, including generative AI, educators can better meet the needs of learners and society. Ultimately, regardless of the variable external pressures and environments, the educator's role to provide a human contact who models adaptability, empathy, and vulnerability remains constant. Although increasing workloads, precarious job security, and competing demands on our time produce a challenging environment for educators, we must remember what a privilege and responsibility it is to help others to learn, to think critically, and to thrive.

## Synthesis

Reviewing Editor: Selva Baltan, Oregon Health &Science University Department of Surgery Decisions are customarily a result of the Reviewing Editor and the peer reviewers coming together and discussing their recommendations until a consensus is reached. When revisions are invited, a fact-based synthesis statement explaining their decision and outlining what is needed to prepare a revision will be listed below. The following reviewer(s) agreed to reveal their identity: NONE.

This is a timely piece that raises awareness of the need for educators to move beyond stagnant, outdated, and dull teaching methods. Constant advances in biosciences demand new teaching approaches that keep up. By leveraging emerging technologies like generative AI, educators can more effectively meet the needs of learners and society. Despite external pressures and challenging environments, the essential role of educators-to foster human connection that shows adaptability, empathy, and vulnerability-remains the same. Although increasing workloads, job insecurity, and multiple demands make teaching difficult, it's important to remember that guiding others to learn, think critically, and succeed is both a privilege and a responsibility.
